# Caffeic acid phenethyl ester alleviates 1,2-dichloroethane-induced toxic cerebral edema: central and peripheral mechanisms

**DOI:** 10.3389/fphar.2025.1734227

**Published:** 2026-01-06

**Authors:** Yue Sun, Bo Yin, Shubei He, Luxin Miao, Jinhan Yang, Yan Wang, Xiaoxia Jin

**Affiliations:** 1 Department of Occupational and Environmental Health, School of Public Health, Shenyang Medical College, Shenyang, Liaoning, China; 2 Department of Forensic Medicine, China Criminal Police University, Shenyang, China; 3 Key Laboratory of Environment and Population Health of the Educational Department of Liaoning Province, Shenyang, China

**Keywords:** 1,2-dichloroethane, CAPE, cerebral edema, inflammatory response, oxidative stress

## Abstract

**Background:**

1,2-Dichloroethane (1,2-DCE) is a widespread environmental contaminant as well as a frequent occupational hazard. Given that inflammation and oxidative stress are key mechanisms in 1,2-DCE-induced cerebral edema, we investigated the efficacy of caffeic acid phenethyl ester (CAPE), a natural anti-inflammatory and antioxidant agent known to protect blood-brain barrier (BBB) integrity, against this intoxication and explored its underlying mechanisms.

**Methods:**

Static inhalation exposure was used to establish a mouse model of 1,2-DCE-induced toxic cerebral edema. Cerebral edema was evaluated based on brain water content, histopathological changes, and tight junction proteins (TJPs) expression. The related anti-inflammatory and antioxidant mechanisms were analyzed by examining the p38 mitogen-activated protein kinase (p38 MAPK) and nuclear factor erythroid 2-related factor 2 (Nrf2) pathways, respectively. Additionally, the levels of specific cytokines and oxidative stress markers were quantified in both brain tissue and serum.

**Results:**

CAPE alleviated the body weight loss and reduced the brain water content in 1,2-DCE-intoxicated mice. Hematoxylin and eosin (HE) staining revealed that CAPE effectively ameliorated the characteristic pathological manifestations of brain edema. CAPE mediated its protective effects through the downregulation of both the p38 MAPK and Nrf2 signaling pathways, resulting in suppressed expression of the cytokines tumor necrosis factor-α (TNF-α), interleukin-1β (IL-1β), and matrix metalloproteinase-9 (MMP-9), normalized levels of glutathione (GSH) and malondialdehyde (MDA), and attenuated loss of the TJPs Occludin and ZO-1. Furthermore, CAPE reversed the 1,2-DCE-induced alterations in pro-inflammatory cytokines and oxidative stress markers in peripheral serum, while inhibiting the expression of vascular cell adhesion molecule-1 (VCAM-1) and intercellular adhesion molecule-1 (ICAM-1) in brain tissue.

**Conclusion:**

This study provides the first evidence that CAPE effectively alleviates cerebral edema through mitigating both peripheral and central inflammatory responses and oxidative stress induced by 1,2-DCE.

## 1 Introduction

1,2-Dichloroethane (1,2-DCE), a highly toxic organic solvent widely used in chemical, pharmaceutical, and electronic manufacturing, has emerged as a prevalent environmental contaminant in the air, drinking water, and soil (Xiang et al., 2023; Zhong et al., 2023). In recent years, severe accidents of subacute 1,2-DCE poisoning have occurred frequently (Chen et al., 2019; Wang et al., 2025). The primary clinical manifestation is toxic encephalopathy, with cerebral edema being the key pathological process, which endangers workers’ health and lives (Li et al., 2025). However, there is still no specific antidote for 1,2-DCE-induced toxic encephalopathy.

Subacute 1,2-DCE poisoning induces mixed cerebral edema, with vasogenic cerebral edema developing initially. As core components of the blood-brain barrier (BBB), tight junction proteins (TJPs) are essential for maintaining its structural integrity and physiological function. Among various TJPs, ZO-1, claudin-5, and Occludin serve as sensitive indicators for assessing BBB permeability (Le Guennec et al., 2025). It has been established that the pro-inflammatory cytokines tumor necrosis factor-α (TNF-α) and interleukin-1β (IL-1β) impair BBB integrity through the downregulation and relocalization of TJPs (Fetsko et al., 2024; Gryka-Marton et al., 2025). Our previous findings demonstrated that subacute 1,2-DCE exposure amplified neuroinflammation and degraded TJPs via the p38 mitogen-activated protein kinase (p38 MAPK) signaling pathway, ultimately promoting cerebral edema (Jin et al., 2018a). Additionally, as an exogenous chemical, 1,2-DCE induced oxidative stress in brain tissue, which in turn exacerbated BBB damage by impairing the expression of TJPs (Jin et al., 2018b). Therefore, pharmacological agents with combined anti-inflammatory, antioxidant, and BBB protectant properties may effectively alleviate 1,2-DCE-induced cerebral edema.

Caffeic acid phenethyl ester (CAPE), a natural bioactive component of propolis, can penetrate the BBB and exhibits notable anti-inflammatory and antioxidant properties. These attributes suggest its potential therapeutic value for preventing and treating brain tissue damage (Kulkarni et al., 2021; Sulimai et al., 2025). Moreover, CAPE preserves BBB integrity by counteracting the loss of claudin-5 expression following brain injury (Zhao et al., 2012). Thus, CAPE can mitigate the occurrence of brain edema through its anti-inflammatory, antioxidant, and TJP-protective effects in the central nervous system. However, unlike prior CAPE research on ischemia, trauma, or oxidative injury models, our experimental model is induced by an exogenous chemical and is explored from both central and peripheral perspectives. Consequently, this study aims to be the first to investigate the protective effect of CAPE against 1,2-DCE-induced toxic brain edema and its underlying mechanisms.

## 2 Materials and methods

### 2.1 Animals and treatment

Female Kunming albino mice (22–24 g, n = 48) were supplied by Liaoning Changsheng Biotechnology Co., Ltd. (Liaoning, China). All mice were maintained under suitable housing conditions with a 1-week acclimatization period prior to experimentation. Animals were randomly assigned to six groups using a random number table (n = 8 per group): control group, CAPE group, 1,2-DCE-exposed group, and three dose-level CAPE intervention groups. Mice in the CAPE control and intervention groups received daily intraperitoneal (i.p.) injections of CAPE (Shanghai Yuanye Biotechnology, China) in 0.2 mL at doses of 25 mg/kg and 1, 5, and 25 mg/kg body weight, respectively. Mice in the control and poisoned groups received daily i.p. injections of dimethyl sulfoxide (DMSO) at equal volumes. Two hours later, mice in the poisoned and intervention groups were subjected to daily static inhalation of 1.2 g/m^3^ 1,2-DCE (analytical reagent; Sinopharm Chemical Reagent, China) for 3.5 h. The detailed operational procedures for establishing the subacute 1,2-DCE exposure model have been described in our previous publication (Wang et al., 2014). After a 3-day intervention and exposure, mice were anesthetized via i.p. injection of 1% sodium pentobarbital (100 mg/kg) and both serum and brain tissues were collected on the fourth day. This study was authorized by Shenyang Medical College’s Ethics Committee.

### 2.2 Brain water content

The standard wet-dry method was used to determine brain water content, as previously described (Sehati et al., 2024). Half of the mouse brain tissue in each group was quickly separated and weighed using an analytical balance to obtain the wet weight. Thereafter, these tissues were dried at 100 °C for 48 h in an oven to obtain the dry weight.

$$\text{Brain water content }\left(\text{expressed as }a\text{ percentage}\right)=\left(\text{wet weight }‐\text{ dry weight}\right)\times 100\%\;/\text{ wet weight}.$$

### 2.3 Western blot analysis

Total protein was extracted with RIPA lysis buffer (Beyotime Biotechnology, China), and its concentration was determined using a bicinchoninic acid (BCA) assay kit (Thermo Fisher Scientific, United States). Proteins (40 µg) were separated, detected and analyzed as previously described (Jin et al., 2019). Primary antibodies used were against matrix metalloproteinase-9 (MMP-9, 1:500, A2095, ABclonal, China), Occludin (1:500, ab167161, Abcam, United States), ZO-1 (1:1000, AB2272, Thermo Fisher Scientific), vascular cell adhesion molecule-1 (VCAM-1, 1:1000, ab134047, Abcam), intercellular adhesion molecule-1 (ICAM-1, 1:1000, ab171123, Abcam), p-p38 MAPK (1:500, AP0526, ABclonal), p38 MAPK (1:1000, A14401, ABclonal, China), phosphorylated nuclear factor-κB (NF-κB) p65 (1:500, AP0475, ABclonal), phosphorylated activator protein-1 (AP-1) c-fos (p-c-fos, 1:500, AP0038, ABclonal), nuclear factor erythroid 2-related factor 2 (Nrf2, 1:1000, A0674, ABclonal), heme oxygenase-1 (HO-1, 1:1000, A1346, ABclonal), and β-actin (served as the internal control, 1:5000, AC026, ABclonal). Secondary antibodies were incubated at a dilution of 1:5000 (AS014 or AS003, ABclonal). Target proteins were visualized using an ECL plus kit (Shanghai Epizyme Biomedical Technology, China).

### 2.4 Histopathological observation

Briefly, mice were deeply anesthetized and transcardially perfused with saline followed by 4% paraformaldehyde (PFA). Brains were fixed in 4% PFA, paraffin-embedded, and sectioned (5-µm-thick coronal sections) for hematoxylin and eosin (HE) staining, as described previously (Yang et al., 2021).

### 2.5 Quantitative real-time RT-PCR assay

Total RNA was extracted from mouse cerebral tissues using Trizol (TaKaRa, Japan), and cDNA was reverse-transcribed with the HiFiScript cDNA synthesis kit (CoWin Biotechnology, China). We amplified transcripts for *Mmp9*, *Ocln*, *Tjp1*, *Mapk14*, *Rela*, *Fos*, *Il1β*, *Tnf*, *Nfe2l2*, *Hmox1*, *Icam1*, and *Vcam1* using the primers detailed in Table 1, with *Gapdh* serving as the internal reference. Quantitative real-time PCR was performed with the UltraSYBR Mixture (CoWin Biotechnology, China). The experimental procedure and data analysis were performed as described previously (Yu et al., 2020).

**TABLE 1:** The primer sequence for PCR.

Gene	Primer sequences (5′→ 3′)		Length (bp)
*Mmp9* (MMP-9)	Sense	GAAGGCTCTGCTGTTCAG	129
	Antisense	AAG​ATG​TCG​TGT​GAG​TTC​C	
*Ocln* (Occludin)	Sense	GCT​ATG​GAG​GCT​ATG​GCT​ATG​G	161
	Antisense	CTA​AGG​AAG​CGA​TGA​AGC​AGA​AG	
*Tjp 1* (ZO-1)	Sense	AAG​CGA​TTC​AGC​AGC​AAC​AG	269
	Antisense	GGA​CCG​TGT​AAT​GGC​AGA​CT	
*Mapk14* (p38 MAPK)	Sense	CGT​TCA​GTT​TCT​CAT​CTA​CC	163
	Antisense	TGT​CAT​CTC​ATC​ATC​AGT​GT	
*Rela* (p65)	Sense	CAC​AGA​TAC​CAC​CAA​GAC​A	155
	Antisense	CAG​CCT​CAT​AGT​AGC​CAT​C	
*Fos* (c-fos)	Sense	CGG​GTT​TCA​ACG​CCG​ACT​A	165
	Antisense	TGG​CAC​TAG​AGA​CGG​ACA​GAT	
*Il1β* (IL-1β)	Sense	GAA​ATG​CCA​CCT​TTT​GAC​AGT​G	116
	Antisense	TGG​ATG​CTC​TCA​TCA​GGA​CAG	
*Tnf* (TNF-α)	Sense	CTG​AAC​TTC​GGG​GTG​ATC​GG	122
	Antisense	GGC​TTG​TCA​CTC​GAA​TTT​TGA​GA	
*Nfe2l2* (Nrf2)	Sense	TTG​GCA​GAG​ACA​TTC​CCA​TTT​G	172
	Antisense	AAA​CTT​GCT​CCA​TGT​CCT​GCT​CTA	
*Hmox1* (HO-1)	Sense	TGC​AGG​TGA​TGC​TGA​CAG​AGG	144
	Antisense	GGG​ATG​AGC​TAG​TGC​TGA​TCT​GG	
*Icam1* (ICAM-1)	Sense	GTG​GGT​CGA​AGG​TGG​TTC​TT	168
	Antisense	GCA​GTT​CCA​GGG​TCT​GGT​TT	
*Vcam1* (VCAM-1)	Sense	CTG​TTC​CAG​CGA​GGG​TCT​AC	287
	Antisense	CAC​AGC​CAA​TAG​CAG​CAC​AC	
*Gapdh* (Gapdh)	Sense	CAA​TGT​GTC​CGT​CGT​GGA​TCT	124
	Antisense	GTC​CTC​AGT​GTA​GCC​CAA​GAT​G	

### 2.6 Cytokine production assay

The TNF-α and IL-1β levels in mouse brain lysates (expressed as pg/mg protein) and serum (expressed as pg/mL) were quantified with commercial ELISA kits (Shanghai Enzyme-linked Biotechnology, China; ml002095A and ml106733A) per the manufacturer’s instructions.

### 2.7 Glutathione (GSH) and malondialdehyde (MDA) levels and SOD activity

Briefly, brain tissues were homogenized. A portion of the homogenate was used for assaying MDA content, while the remainder was centrifuged to collect the supernatant for SOD and GSH analysis with commercial kits (Nanjing Jiancheng Bioengineering, China; A003-1, A001-3, and A006-2-1). Additionally, the serum was pretreated as directed by the manufacturer prior to the measurement of these indicators. BCA protein assay kits were used to evaluate the protein concentrations.

### 2.8 Statistical analysis

Data were presented as mean ± standard deviation (SD). Statistical analyses were conducted using SPSS software (version 22.0; SPSS Inc., IL, United States). The significant differences among groups were evaluated by one-way analysis of variance (ANOVA) followed by the least significant difference (LSD) test. A *P* value <0.05 was considered statistically significant.

## 3 Results

### 3.1 Effect of CAPE on general health in mice with subacute 1,2-DCE exposure

**FIGURE 1:**
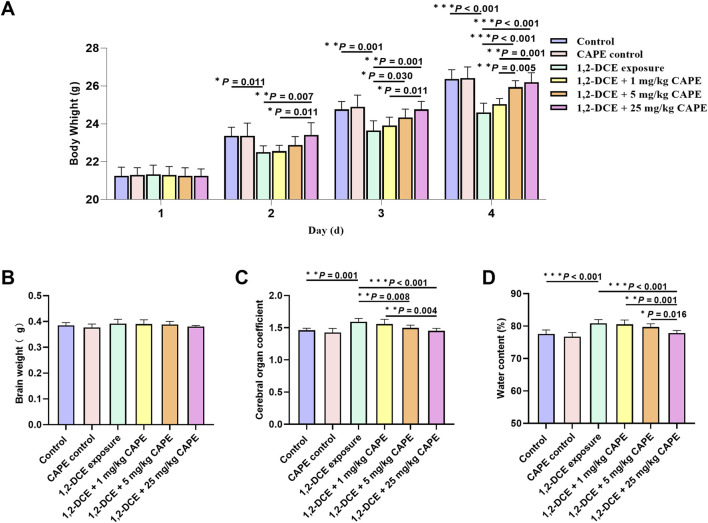
Effect of CAPE on general health in mice with subacute 1,2-DCE exposure. **(A)** Changes in body weight of mice in each group throughout the experiment. **(B–D)** Comparison of brain weight, cerebral organ coefficient and water content among groups. Results were represented as mean ± SD, n = 5. Statistical significance was evaluated using one-way ANOVA followed by the LSD test. *P < 0.05; **P < 0.01; ***P < 0.001.

Over the course of exposure, body weight increased in all groups. Starting from the second day of the experiment, mice in the 1,2-DCE-exposed group showed a marked decrease in body weight compared to the control group. On the second day of the experiment, CAPE intervention at 25 mg/kg significantly reversed the body weight loss induced by 1,2-DCE; on the third and fourth days, both the 5 and 25 mg/kg doses of CAPE effectively restored body weight (Figure 1A). No significant difference in brain weight was observed across the groups (Figure 1B). Compared with the control group, 1,2-DCE-exposed mice exhibited a significant increase in both the brain organ coefficient and cerebral water content, which were ameliorated to varying degrees by CAPE intervention (Figures 1C,D).

### 3.2 CAPE ameliorates brain edema and associated neuropathology

**FIGURE 2:**
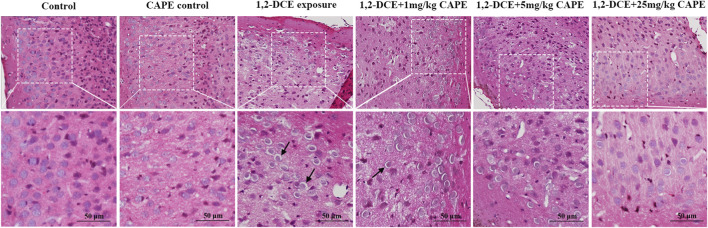
CAPE ameliorates brain edema and associated neuropathology. Representative HE-stained sections of brain tissue (×400 magnification, scale bar = 50 μm, n = 3 per group). The arrow indicates the enlarged perinuclear space.

Brain tissues from the control mice exhibited intact cytoarchitecture without pathological alterations. In contrast, 1,2-DCE exposure induced typical pathological changes of cytotoxic cerebral edema, with loosened intercellular substance, widened perinuclear vacuolization, swollen cell bodies, and blurred cellular boundaries. These neuropathological changes were markedly improved by 25 mg/kg CAPE intervention (Figure 2).

### 3.3 CAPE attenuates MMP-9 and restores TJPs in brain of 1,2-DCE-exposed mice

**FIGURE 3:**
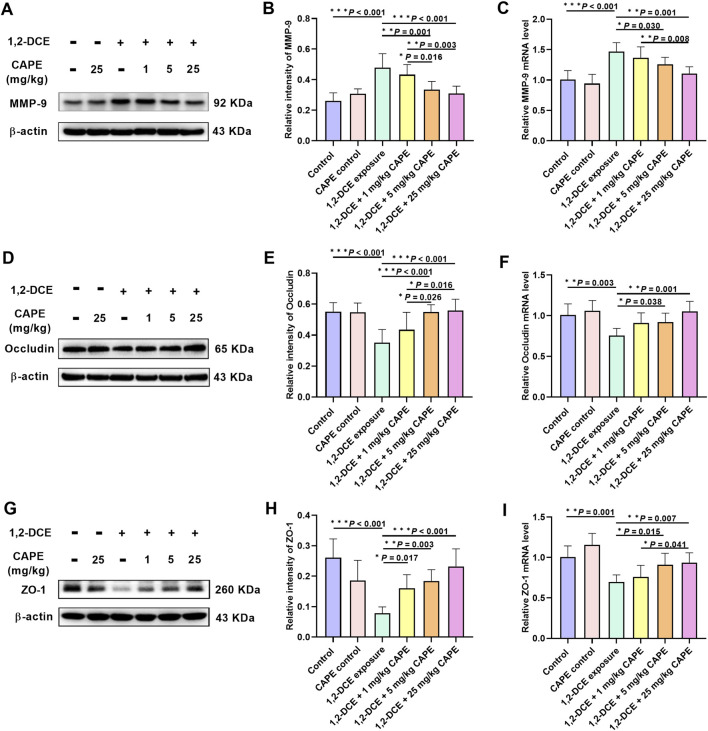
CAPE attenuates MMP-9 and restores TJPs in brain of 1,2-DCE–exposed mice. **(A–I)** The protein and gene expression levels of MMP-9, Occludin and ZO-1 were measured by Western blot and real-time RT-PCR, respectively, and the images shown are representative blots. Results were represented as mean ± SD, n = 5. Statistical significance was evaluated using one-way ANOVA followed by the LSD test. *P < 0.05; **P < 0.01; ***P < 0.001.

In the 1,2-DCE-exposed group, MMP-9 expression was significantly upregulated, whereas the expression of Occludin and ZO-1 was downregulated. Compared with the 1,2-DCE-exposed group, 5 and 25 mg/kg CAPE intervention significantly suppressed MMP-9 expression at both transcriptional and translational levels. Additionally, CAPE improved the expression levels of ZO-1 and Occludin to varying degrees, with 25 mg/kg CAPE exerting a more pronounced effect at both mRNA and protein levels (Figure 3).

### 3.4 CAPE attenuates neuroinflammatory response in 1,2-DCE-induced cerebral edema

**FIGURE 4:**
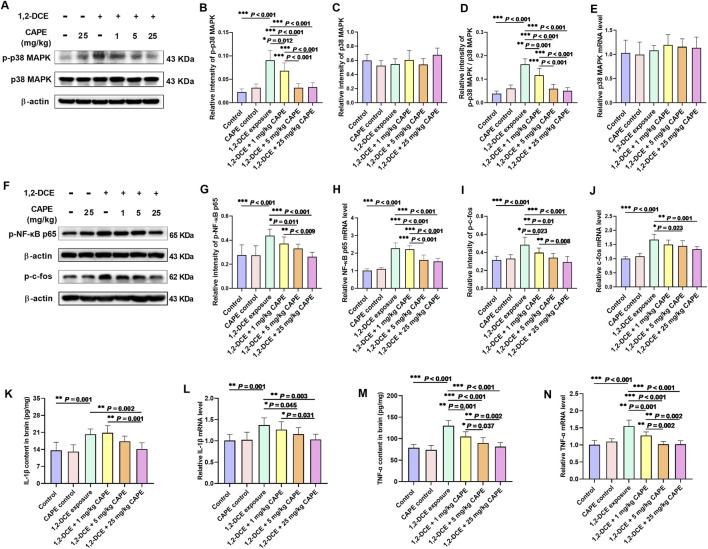
CAPE attenuates neuroinflammatory response in 1,2-DCE-induced cerebral edema. **(A–C)** The protein expression levels of p38 MAPK and p-p38 MAPK were measured by Western blot, and the images shown are representative blots. **(D)** Levels of p-p38 MAPK were normalized to the intensity of native p38 MAPK, expressed as p-p38 MAPK/p38 MAPK. **(E)** The mRNA expression levels of *Mapk14*. **(F–J)** The protein and gene expression levels of phosphorylated NF-kB (p65) and AP-1 (c-fos) were measured by Western blot and real-time RT-PCR, respectively, and the images shown are representative blots. **(K–N)** The content and mRNA expression levels of proinflammatory cytokines TNF-α and IL-1β in the brain tissue. Results were represented as mean ± SD, n = 5. Statistical significance was evaluated using one-way ANOVA followed by the LSD test. *P < 0.05; **P < 0.01; ***P < 0.001.

All doses of CAPE intervention significantly reduced the 1,2-DCE-induced upregulation of phosphorylated p38 MAPK expression, showing a dose-dependent relationship (Figures 4A–E). Compared to the 1,2-DCE-exposed group, CAPE intervention markedly inhibited the expression of downstream nuclear transcription factors AP-1 (c-fos) and NF-κB (p65) at both the gene transcription and phosphorylation levels. Among these, p-c-fos was more sensitive to CAPE intervention (Figures 4F–J). In addition, CAPE intervention dose-dependently attenuated the expression of TNF-α and IL-1β to varying extents in the brain tissue of 1,2-DCE-exposed mice (Figures 4K–N).

### 3.5 CAPE mitigates oxidative stress in 1,2-DCE-caused toxic brain edema

**FIGURE 5:**
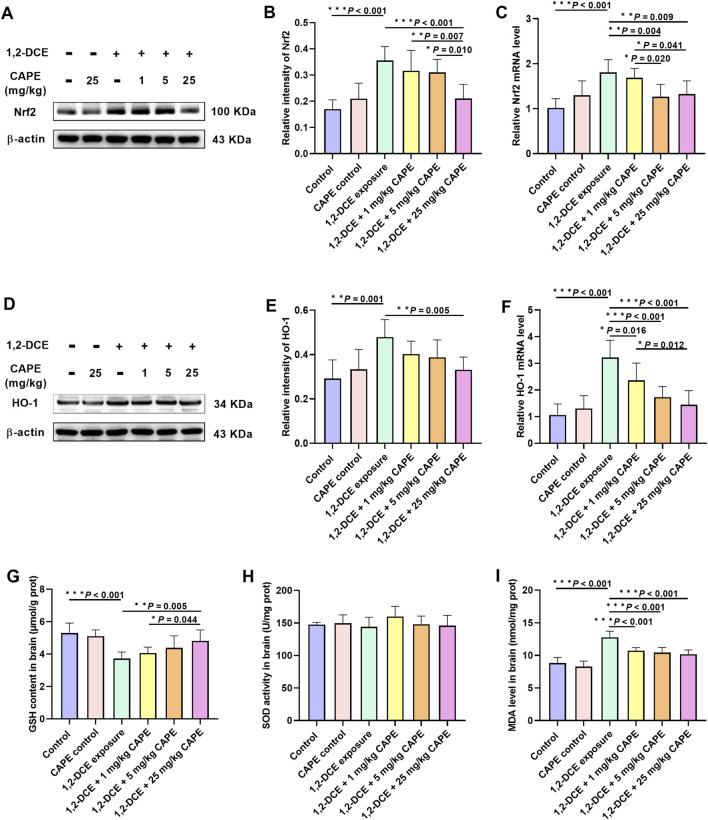
CAPE mitigates oxidative stress in 1,2-DCE-caused toxic brain edema. **(A–F)** The protein and mRNA expression levels of Nrf2 and HO-1 were measured by Western blot and real-time RT-PCR, respectively. The images shown are representative blots. **(G–I)** The content of GSH and MDA and the activity of SOD in brain tissue. Results were represented as mean ± SD, n = 5. Statistical significance was evaluated using one-way ANOVA followed by the LSD test. *P < 0.05; **P < 0.01; ***P < 0.001.

Compared with the control group, exposure to 1,2-DCE upregulated the expression of Nrf2 and HO-1, increased MDA content, and decreased the level of the antioxidant substance GSH in brain tissue. In contrast to the 1,2-DCE-exposed group, CAPE intervention significantly downregulated the expression of Nrf2 and HO-1 to varying degrees, with transcriptional regulation playing a predominant role (Figures 5A–F). In addition, CAPE attenuated the changes in GSH and MDA content in brain tissue caused by 1,2-DCE. However, there was no significant change in SOD activity among the groups (Figures 5G–I).

### 3.6 Peripheral effects of CAPE on 1,2-DCE-induced brain edema

**FIGURE 6:**
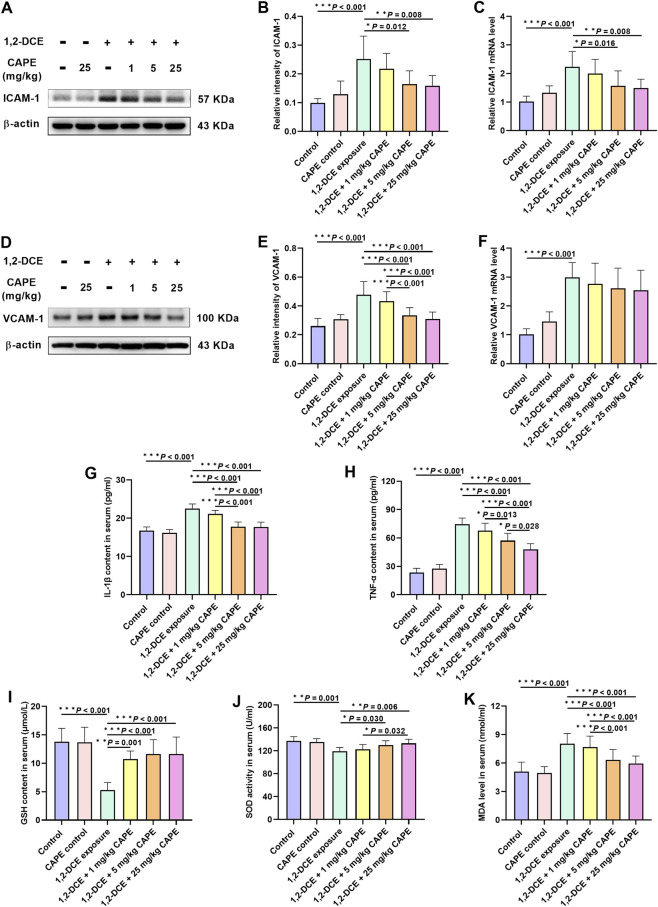
Peripheral effects of CAPE on 1,2-DCE-induced brain edema. **(A–F)** The protein and mRNA expression levels of VCAM-1 and ICAM-1 were measured by Western blot and real-time RT-PCR, respectively. The images shown are representative blots. **(G,H)** The content of proinflammatory cytokines TNF-α and IL-1β in serum. **(I–K)** The content of GSH and MDA and the activity of SOD in serum. Results were represented as mean ± SD, n = 5. Statistical significance was evaluated using one-way ANOVA followed by the LSD test. *P < 0.05; **P < 0.01; ***P < 0.001.

Exposure to 1,2-DCE upregulated the expression of VCAM-1 and ICAM-1. Both 5 and 25 mg/kg CAPE downregulated ICAM-1 expression at the transcriptional and translational levels, whereas VCAM-1 expression was inhibited only at the protein level (Figures 6A–F).

Given that VCAM-1 and ICAM-1 are expressed on the vascular side of BBB endothelial cells, we measured changes in pro-inflammatory cytokines and oxidative stress markers in serum to explore their potential contribution. Results showed that intervention with 5 and 25 mg/kg CAPE significantly reduced the levels of TNF-α and IL-1β, as well as the content of the oxidative damage marker MDA, while upregulating SOD activity. All doses of CAPE intervention significantly increased GSH content (Figures 6G–K).

## 4 Discussion

### 4.1 CAPE protects against 1,2-DCE-induced toxic cerebral edema

Toxic encephalopathy is the most common and serious consequence of 1,2-DCE exposure, with cerebral edema serving as its main pathological manifestation (Wang et al., 2025; Xiang et al., 2023). In this study, a mouse model of 1,2-DCE-induced toxic cerebral edema was successfully established, as confirmed by body weight loss, increased brain water content and brain organ coefficient, and characteristic pathological changes in mice. CAPE intervention alleviated these alterations, demonstrating its protective effect against 1,2-DCE-induced toxic cerebral edema.

### 4.2 CAPE preserves BBB integrity by regulating MMP-9 and TJPs

It is well established that BBB disruption allows toxins and pathogens to infiltrate brain tissue and cause cerebral edema (Khor et al., 2024). Our earlier work showed that overexpression of the protease MMP-9 degraded TJPs and compromised BBB integrity, constituting a primary mechanism in the early phase of 1,2-DCE poisoning (Jin et al., 2019). Given that CAPE maintains TJP levels by regulating MMP-9 (Lu et al., 2022), we examined whether it acted similarly in this model. Our results confirmed that CAPE alleviated brain edema by restoring the expression of TJPs ZO-1 and Occludin at both transcriptional and translational levels through downregulation of MMP-9. Further observation revealed that ZO-1 was more responsive to CAPE intervention, an effect that might occur through pathways other than the MMP-9 degradation in 1,2-DCE poisoning. Building on our previous finding that MMP-9 expression is regulated by the p38 MAPK pathway (Jin et al., 2018a), we next investigated how CAPE modulated this pathway.

### 4.3 CAPE suppresses neuroinflammation in 1,2-DCE intoxication via the p38 MAPK pathway

The p38 MAPK inflammatory pathway significantly contributes to cerebral edema pathogenesis (Chen et al., 2024; Zhu et al., 2024). Our results indicated that CAPE intervention selectively suppressed the phosphorylation of p38 MAPK induced by 1,2-DCE, without affecting its gene expression or total protein levels. NF-κB and AP-1 act as pivotal downstream nuclear transcription factors of the inflammatory p38 MAPK pathway implicated in brain edema (Jin et al., 2018a). The predominant NF-κB dimer is p50-p65, and the phosphorylated modification of p65 is a critical marker for evaluating NF-κB activation (Shen et al., 2025). Our findings indicated that CAPE inhibited 1,2-DCE-induced NF-κB activation by reducing both p65 transcription and phosphorylation levels. As an immediate-early gene, the phosphorylation level of c-fos reflects the activation status of AP-1 (Takashina et al., 2018). Our results demonstrate that CAPE inhibits 1,2-DCE-induced AP-1 activation mainly by decreasing c-fos phosphorylation rather than its transcription. Moreover, AP-1 and NF-κB can directly regulate the expression of pro-inflammatory cytokines such as IL-1β and TNF-α (Abd-Elhamid et al., 2024; da Silva et al., 2025). Under pathological conditions, the sustained overproduction of TNF-α and IL-1β can alter BBB permeability and exacerbate cerebral edema (Versele et al., 2022). In this study, CAPE intervention significantly decreased the overexpression of TNF-α and IL-1β, with TNF-α being more sensitive. By analyzing the minimal effective doses for the aforementioned indicators, together with our previous conclusions, we propose that CAPE ameliorates 1,2-DCE-induced toxic brain edema by suppressing the inflammatory response via the p38 MAPK pathway.

### 4.4 CAPE alleviates oxidative stress and modulates the Nrf2/HO-1 pathway

When ROS production exceeds the body’s clearance capacity, it induces lipid peroxidation in the BBB, thereby disrupting BBB integrity and aggravating brain edema (Robinson, et al., 2025). Accordingly, three classical oxidative stress biomarkers were detected: the antioxidant enzyme SOD and the non-enzymatic antioxidant GSH, both of which are major free radical scavengers and serve as reliable indicators of systemic antioxidant capacity; and the lipid peroxidation product MDA, which provides direct evidence of oxidative damage (Amini et al., 2023). 1,2-DCE intoxication significantly decreased GSH levels and markedly increased MDA levels in brain tissue, without altering SOD activity. CAPE intervention reversed these changes, indicating its antioxidant effect against 1,2-DCE-induced toxic brain edema.

Moreover, as the core regulatory hub of the endogenous antioxidant system, the Nrf2/HO-1 pathway mitigates brain edema by counteracting oxidative damage (Liu et al., 2022). However, 1,2-DCE exposure unexpectedly upregulated Nrf2 and HO-1 in the present model, contrary to some reports (Luo et al., 2025; Mehanna et al., 2025). A previous study has confirmed that initial oxidative stress caused by exogenous toxins triggers compensatory Nrf2 and HO-1 activation (Wei et al., 2025), while sustained exposure leads to antioxidant depletion and eventually suppresses their expression (Abulikemu et al., 2023). Therefore, during subacute 1,2-DCE poisoning in mice, the body may enter a compensatory antioxidant phase with limited protective efficacy; correspondingly, CAPE intervention alleviates oxidative damage while simultaneously suppressing this compensatory response.

### 4.5 CAPE reduces cerebral edema by attenuating peripheral inflammation and oxidative stress

On the other hand, the upregulation of VCAM-1 and ICAM-1 on the vascular endothelial surface is closely associated with BBB disruption (Alshammari et al., 2024). ICAM-1 increases vascular permeability and disrupts the BBB structure by facilitating stable neutrophil adhesion to the endothelium (Yu et al., 2022). VCAM-1 promotes the migration of peripheral inflammatory cells into brain lesions by enhancing their adhesion, thereby exacerbating neuroinflammation (Gao et al., 2021). In this study, 1,2-DCE exposure increased VCAM-1 and ICAM-1 expression in the brain, consistent with previous research (Yang et al., 2021). Notably, CAPE intervention significantly counteracted these specific changes. Prior research has demonstrated that alterations in peripheral conditions can exacerbate BBB destruction by modulating VCAM-1 and ICAM-1 expression (Cook-Mills et al., 2011; Rodriguez et al., 2025). We therefore measured changes in pro-inflammatory cytokines and oxidative damage markers in serum. Our results indicate that CAPE markedly decreased the levels of TNF-α, IL-1β and MDA, increased GSH levels, and enhanced SOD activity. Overall, these findings showed that CAPE significantly attenuated peripheral inflammation and enhanced peripheral antioxidant capacity, suggesting its potential to mitigate vasogenic cerebral edema by modulating the peripheral environment to reduce adhesion molecule expression.

### 4.6 Limitations and future perspectives

Suppressing peripheral inflammation and oxidative stress helps maintain BBB integrity (Farias et al., 2025; Yoon, et al., 2025). Nevertheless, the mechanisms through which CAPE ameliorates cerebral edema from a peripheral perspective may involve more than the regulation of VCAM-1 and ICAM-1. Therefore, the specific protective mechanism of CAPE in 1,2-DCE-poisoned mice warrants further investigation. Moreover, for subsequent studies, CAPE dosing should be aligned with human exposure levels to improve its translational potential.

## 5 Conclusion

**FIGURE 7:**
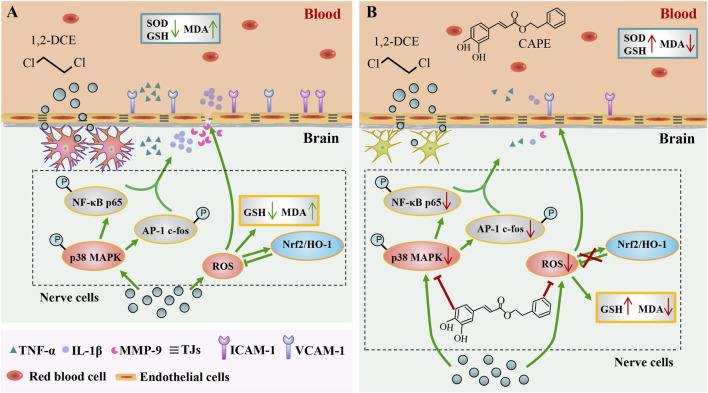
Schematic diagram. **(A)** 1,2-DCE-induced toxic pathway: Upon entering the systemic circulation, 1,2-DCE triggered peripheral inflammation and oxidative stress. This was characterized by elevated pro-inflammatory cytokines TNF-α and IL-1β, depletion of the antioxidant GSH, inhibition of the activity of the antioxidant enzyme SOD, and accumulation of the lipid peroxidation product MDA. Consequently, the expression of vascular adhesion molecules ICAM-1 and VCAM-1 was upregulated. Moreover, upon crossing the BBB, 1,2-DCE activated the p38 MAPK pathway in the brain, upregulating TNF-α, IL-1β, and MMP-9 to amplify neuroinflammation, which ultimately downregulated tight junction proteins and disrupted the BBB. Although 1,2-DCE exposure also induced a compensatory activation of the Nrf2 pathway in the brain, the concurrent accumulation of MDA and depletion of GSH demonstrated the insufficiency of this protective response to counteract oxidative stress, ultimately exacerbating cerebral edema. **(B)** The protective effect of CAPE: Through counteracting the aforementioned adverse effects in both the periphery and the central nervous system, CAPE intervention effectively preserved BBB integrity and alleviated cerebral edema.

This study provides the first investigation into the protective effect of CAPE against 1,2-DCE-induced toxic cerebral edema in mice. Unlike previous research, our work specifically elucidates how CAPE alleviates brain edema from both central and peripheral viewpoints. The results showed that CAPE suppressed adhesion molecule expression via its peripheral anti-inflammatory and antioxidant effects, while it also preserved TJPs and maintained BBB integrity by modulating the p38 MAPK and Nrf2 signaling pathways in the brain (Figure 7). These results highlight CAPE’s potential as a promising therapeutic candidate for the prevention and treatment of occupational 1,2-dichloroethane poisoning.

## Data Availability

The raw data supporting the conclusions of this article will be made available by the authors, without undue reservation.
